# Detection and Genomic Characterization of Bovine Rhinitis Virus in China

**DOI:** 10.3390/ani13020312

**Published:** 2023-01-16

**Authors:** Yuxing Zhou, Xi Chen, Cheng Tang, Hua Yue

**Affiliations:** 1 College of Animal and Veterinary Sciences, Southwest Minzu University, Chengdu 610041, China; 2 Key Laboratory of Ministry of Education and Sichuan Province for Qinghai-Tibetan Plateau Animal Genetic Resource Reservation and Utilization, Chengdu 610041, China

**Keywords:** bovine rhinitis A virus, bovine rhinitis B virus, prevalence, genomes, recombination

## Abstract

**Simple Summary:**

Bovine rhinitis virus (BRV) is an important pathogen causing bovine respiratory disease complex (BRDC) and it can be divided into two genotypes—bovine rhinitis A virus (BRAV) and bovine rhinitis B virus (BRBV). However, knowledge about the prevalence and molecular information of BRV in China is still limited. The purpose of this study was to investigate the prevalence and molecular characteristics of BRV in beef cattle with BRDC syndrome in China, and the results confirmed the circulation of BRAV and BRBV. The genomic sequences of BRAVs in this study showed an independent evolutionary trend while BRBVs exhibited significant genetic heterogeneity, and the recombination patterns of both types of BRVs were distinct from what was observed in a previous study. The present study reported the presence and prevalence of BRAV in China, providing recommendations for prevention and control of BRDC, and contributed to a better understanding of the evolution of BRV.

**Abstract:**

Bovine rhinitis virus (BRV) is an etiological agent of bovine respiratory disease complex (BRDC) and can be divided into two genotypes—bovine rhinitis A virus (BRAV) and bovine rhinitis B virus (BRBV). However, knowledge about the prevalence and molecular information of BRV in China is still limited. In this study, 163 deep nasal swabs collected from bovines with BRDC syndrome on 16 farms across nine provinces of China were tested for BRAV and BRBV by a duplex real-time RT-PCR assay. The results showed that 28.22% (46/163) of the samples were BRV-positive, and the positive rates were 22.09% (36/163) for BRAV and 9.2% (15/163) for BRBV. The co-circulation of both BRV genotypes was observed on two farms. Furthermore, five near-complete BRV genomes, including three BRAVs and two BRBVs, were obtained. The phylogenetic analysis showed that the three obtained BRAVs were phylogenetically independent, while the two BRBVs exhibited significant genetic heterogeneity. Recombination analysis revealed that three BRAVs and one BRBV strain obtained in this study were recombinants. The present study confirmed the presence and prevalence of BRAV in China, and it found that both types of BRV are circulating in beef cattle, which contributes to a better understanding of the prevalence and molecular characteristics of BRV.

## 1. Introduction

Bovine rhinitis virus (BRV) is a non-enveloped, single-stranded, positive-sense RNA virus and belongs to the genus Aphthovirus, family Picornaviridae, and order Picornavirales, and it is an etiological agent of bovine respiratory disease complex (BRDC) [1,2,3] that damages turbinate, trachea, and lung epithelium [1,2,4]. BRV can be divided into bovine rhinitis A virus (BRAV) and bovine rhinitis B virus (BRBV) according to the genomic characterization [5]; of these two genotypes, BRAV is reported to be more virulent than BRBV [6]. To date, the virus has been identified in ten countries across five continents, including Germany, England, Japan, USA, Mexico, Canada, Sudan, Sweden, China, and Australia [7], and has caused significant losses in certain countries [6,8].

At present, there are 20 BRAV and 26 BRBV genomic sequences from nine countries in the GenBank database, and BRBV_CHN1 (Accession Number: MT160419) is the sole Chinese BRV sequence in the database [9]. The genome of BRV contains two untranslated regions (UTRs), four structural protein genes (VP1, VP2, VP3, and VP4), and eight non-structural protein genes (Leader, 2A, 2B, 2C, 3A, 3B, 3C, and 3D). Although little is known about the precise functions of the proteins in BRV, many findings for other Aphthoviruses might also provide implications for BRV. The VP1, VP2, and VP3 proteins make up the outer surface of the capsid, and VP1 is the most external and immunodominant subunit [10,11], while VP4 is embedded within the inner surface of the viral capsid [12]. The Leader and 2A protein induce suppression of host protein synthesis by their protease activity [13,14], and the 2B protein forms pores in the host cell membrane to aid in viral budding and invading [15], which also blocks the host protein synthesis with the help of the 2C protein [16]. The 3A protein plays an important role in determining host range [17,18,19] and the 3B protein of Picornavirus initiates the RNA synthesis [20]. The 3C proteinase cleaves most of the junctions within the viral polyprotein [21], and the 3D protein (3D_pol_), also known as RNA dependent RNA polymerase (RDRP), catalyzes genome replication and withstands high selective pressures [22]. Homologous recombination is a fundamental evolutionary mechanism for Aphthovirus [23], which has been reported to increase the genetic diversity of BRV [22].

Recently, Xie et al. reported an 11% positive rate for BRBV in three southeastern provinces in China [24], and a BRBV genome was further obtained and analyzed [9]. However, knowledge about the prevalence and molecular information of BRV in China isstill very limited, and information regarding BRAV still remains unreported. The aim of this study was to investigate the prevalence and the molecular features of BRVs in China. Here, both BRAV and BRBV were found circulating in some provinces, and the co-circulation of both genotypes were observed on some beef cattle farms. Moreover, the BRAVs that were found exhibited independent evolutionary patterns, while the BRBVs presented significant genetic heterogeneity and distinct evolutionary lineages.

## 2. Materials and Methods

### 2.1. Samples

A total of 163 deep nasal swabs from 16 beef cattle farms (in nine provinces) with BRDC outbreaks were collected from November 2020 to January 2022. Detailed sample information is provided in Table 1. The beef cattle were two to six months old with symptoms characterized by fever, cough, runny nose, dyspnea, etc. All samples were transferred with dry ice and stored at −80 °C for further use.

### 2.2. RNA Extraction and cDNA Synthesis

For each nasal swab sample, 5 mL DMEM was added with a 30s vortexing and it was passed through a 0.22-µm filter. Viral RNA was extracted from 300 µL of the supernatants using RNAios Plus (TaKaRa, Dalian, China) and prepared into single-stranded cDNA using PrimeScript RT Reagent (TaKaRa, Dalian, China) according to the manufacturer’s instructions.

### 2.3. Detection of BRAV and BRBV

A duplex real-time RT-PCR assay for simultaneously detecting BRAV and BRBV established in our previous study was used to detect BRV. The specificity and stability of the assay had been verified, and the lower limits of detection were 32 copies/μL for both BRAV and BRBV. The primer and probe sequences were as follows: F-BRAV, 5′-GTGGGAGACTAAGGATGCC-3′; R-BRAV, 5′-GGAAAAGGCCCGGC-3′; Probe-BRAV, VIC-5′-AGGTAACAAGTGACACTCTGGATCTGAC-3′-BHQ2; F-BRBV, 5′-TGAGATGGGTACACAAAATGTC-3′; R-BRBV, 5′-ATTGTGCCGAATTGTCCC-3′; Probe-BRBV, FAM-5′-TGAATTGAACATAACCAAACTAACTCCAG-3′-BHQ2. The 20 μL system was used, including 10 µL of Premix Ex Taq (Probe qPCR) (Takara, Dalian, China), 1.5 µL cDNA sample, 1 µL of each BRAV primer (10 μM), 3 µL of each BRBV primer (10 μM), and 0.25 µL of each TaqMan probe (10 μM). The reaction conditions were as follows: 2 min pre-denaturation at 95 °C and 40 cycles of (95 °C denaturation for 15 s, 50.2 °C annealing and extension for 20 s).The result was judged as positive when the cycle threshold value was lower than 35.

### 2.4. Genome Amplification

To amplify BRV genomes, nine pairs of BRAV primers and eight pairs of BRBV primers were designed using the Primer Premier 5.0 program for this study and they are shown in Table 2. The reaction conditions were as follows: 5 min pre-denaturation at 95 °C, followed by 35 cycles of (95 °C denaturation for 30 s, annealing according to respective temperatures in Table 2 for 30 s, 72 °C extension for 1 min), ending with a 7-min extension at 72 °C (Takara rTaq Kit, TaKaRa, Dalian, China).All amplified products were purified and cloned into pMD19-T vector (TaKaRa, Dalian, China) for Sanger sequencing (TSING KE Biological Technology, Beijing, China). The sequencing reads were assembled using the SeqMan program, and the correctness of the contigs were verified by additional PCR amplification and Sanger sequencing.

### 2.5. Sequence, Phylogenetic, and Recombination Analysis

Sequence identities were calculated using the MegAlign program in both nucleotide (nt) and amino acid (aa) levels. Evolutionary models were tested and the best model was further used to build phylogenetic trees withIQtree1.6.12. The tree topologies were validated by 1000 replicates of bootstrap resampling. Recombination analyses were performed using RDP, GeneConv, Chimaera, MaxChi, BootScan, SiScan, 3Seq, and Phylpro algorithms in RDP 4.101, and recombination events supported by at least five algorithms were considered reliable.

### 2.6. GenBank Sequence Submission

The obtained nucleotide sequences were deposited in the GenBank database under accession numbers MZ004694~MZ004696 for BRAV CHN1_LZ01, CHN1_LZ10, and CHN1_LZ11; and MZ052127~MZ052128 for BRBV SC1_LZ02 and SC1_LZ05.

## 3. Results

### 3.1. Detection of BRAV and BRBV

**Figure 1:**
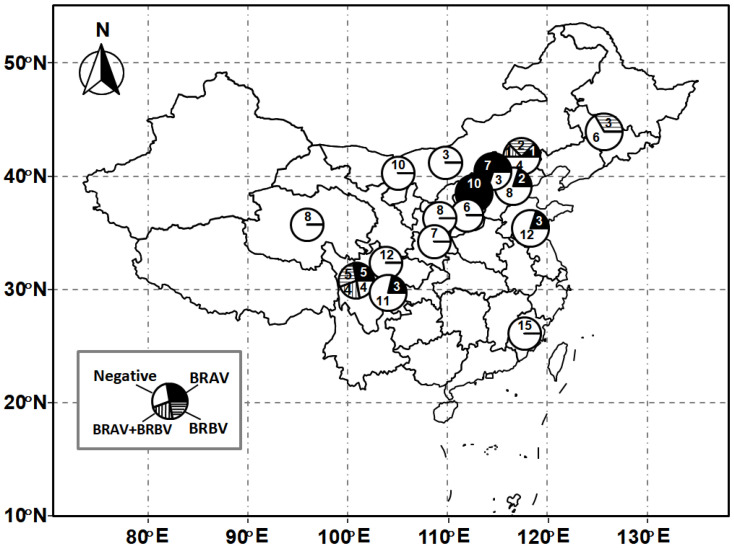
Geographical distribution of samples. Numbers of samples were noted in each pie chart.

Out of 163 nasal swabs collected from bovines with BRDC syndrome, 46 samples (28.22%) from eight farms (50%) were detected as positive for BRV. Of these, 36 samples (22.09%) were positive for BRAV, and 15 (9.2%) samples were positive for BRBV. Out of 16 beef cattle farms, seven farms were detected as positive for BRAV, and three farms were detected as positive for BRBV. Five out of nine investigated provinces, including Sichuan, Shanxi, Hebei, Jilin, and Jiangsu provinces, were positive for BRV (Table 1). The geographical distribution of the samples is depicted in Figure 1. Interestingly, co-infection with both types of BRV was detected in five cattle from two farms (Figure 1).

### 3.2. Molecular Characterization of BRAV Sequences

**Figure 2:**
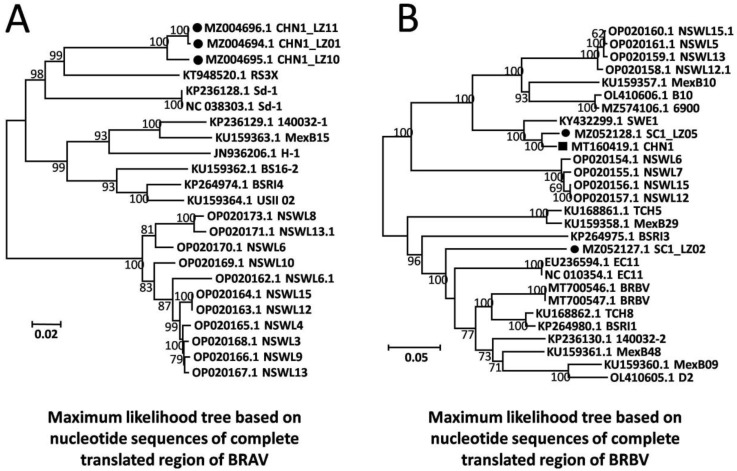
Maximum likelihood trees based on nucleotide sequences of complete translated region of BRV. (**A**) Maximum likelihood trees of BRAV. (**B**) Maximum likelihood trees of BRBV. Solid circles represent BRVs in this study; solid rectangle represents the Chinese BRV strain available in GenBank. The tree topologies were validated by 1000 replicates of bootstrap resampling.

A total of three near-full-length BRAV genomes were obtained from the beef cattle farm in Sichuan province with the highest mortality rate, and these were named CHN1_LZ01 (GenBank Accession: MZ004694), CHN1_LZ10 (GenBank Accession: MZ004695), and CHN1_LZ11 (GenBank Accession: MZ004696). The three genomic sequences were 6951 nt in length, sharing 97.3–99.9% nt and 99.5–99.9% aa sequence identity with each other, and the G+C contents were 51.0%, 50.5%, and 51.1%, respectively. Compared to all the other 20 available BRAV genomes in the GenBank database, the three obtained BRAVs shared 79.3–87.5% nt sequence identity and 90.8–98.4% aa sequence identity. Based on nucleotide sequences of the complete translated region, a phylogenetic tree was built by maximum likelihood algorithm for all 20 available BRAV genomic sequences in GenBank along with the three obtained BRAVs under the best evolutionary model SYM+I+G4, and the results showed that the three obtained BRAVs formed an independent branch (Figure 2A). Furthermore, phylogenetic analysis based on all individual genes obtained consistent results and revealed that the three BRAVs formed unique branches (Supplementary Materials), showing the independent evolutionary trend of the BRAVs in this study.

Alignment analysis of aa sequences showed that the three obtained BRAVs shared 11 unique aa mutations compared with the other 20 available BRAV genomes in GenBank, and these were located in Leader (SRNK17T), VP3 (TENSQ631A), VP1 (GS905A), 2A (V964I), 2B (EA1091T), 3A (L1419Q, K1488R), 3C (SN1708G, I1716V), and 3D_pol_ (TERK1891G, A1923V) referring to the three BRAV sequences obtained in this study (GenBank Accession: MZ004694-MZ004696). The multiple sequence alignment (MSA) plots can be found in the Supplementary Materials.

**Figure 3:**
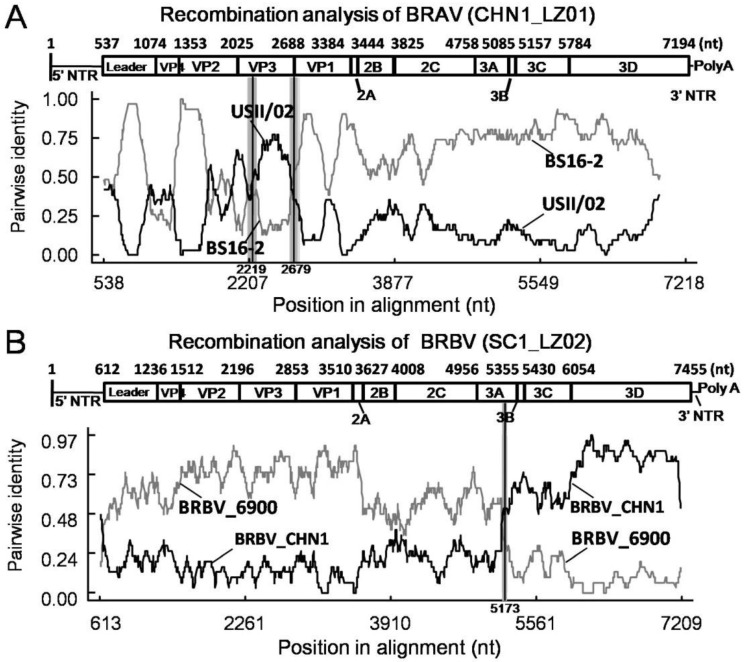
Recombination analysis of BRAV and BRBV. (**A**) Pairwise identity of recombinant BRAV CHN1_LZ01 with major parent BS16-2 and minor parent USII/02. (**B**) Pairwise identity of recombinant BRBV SC1_LZ02 with major parent BRBV_6900 and minor parent BRBV_CHN1.

Next, RDP 4.101 was used to perform recombination analysis of these BRAV sequences. Interestingly, identical recombination events were found in the three BRAVs in this study with a recombinant score of 0.646, which was supported by seven algorithms including RDP, GENECONV, BootScan, MaxChi, Chimaera, SiScan, and 3Seq. The breakpoints were determined beginning at nt 2219 and ending at nt 2679 in the whole genome, and the recombinant region was located in VP3 gene as shown in Figure 3 (which only showsCHN1_LZ01 as representative strain since the three BRAVs shared identical recombination events). The putative major parental strain was BS16-2 (GenBank Accession: KU159362), and the minor parental strain was USII/02 (GenBank Accession: KU159364).

### 3.3. Molecular Characterization of BRBV Sequences

A total of two near-full-length BRBV genomes (SC1_LZ02 and SC1_LZ05, GenBank Accession: MZ052127-MZ052128) were obtained from the beef cattle farm in Sichuan province with the highest mortality rate. The lengths of the two genomes were 7472 and 7423 nt, and the G+C contents were 46.3% and 46.4%, respectively. The two genomes shared 79.2% nt and 85.3% aa sequence identity with each other, exhibiting significant genetic heterogeneity, and shared 73.4–97.1% nt and 83.1–99.3% aa sequence identity with all the 26 BRBV genomes available in the GenBank database. Of the two genomes, SC1_LZ05 shared 97.1% nt and 99.3% aa sequence identity with the Chinese strain BRBV_CHN1 (GenBank Accession: MT160419) and might be its ortholog; SC1_LZ02 shared 73.4–85.6% nt and 83.1–93.2% aa sequence identity with the other 26 BRBV genomes in GenBank, exhibiting significant divergence from all available BRBV genomes, and it shared the highest sequence identity (85.6% nt and 93.2% aa identity) with English strain EC11 (GenBank Accession: NC_010354). Based on nucleotide sequences of the complete translated region, a phylogenetic tree was built by maximum likelihood algorithm for all 26 BRBV genomic sequences under the best evolutionary model TIM2+F+I+G4, and the results showed that SC1_LZ02 formed an independent clade adjacent to English strain EC11, while SC1_LZ05 was located on another branch with Chinese strain BRBV_CHN1 (Figure 2B).

Of the two BRBV strains in this study, SC1_LZ05 contained five unique aa mutations compared with all the 26 BRBV genomes in the GenBank database, and they were located in the 2C (AS1359T), 3C (TS1650N, IT1706A, I1763V), and 3D_pol_ (V2042I), referring to SC1_LZ05; however, SC1_LZ02 showed 33 unique aa mutations located in structural proteins including the VP1 (RT762S, D777E, AKMSE789G, TNKRH801Q, ATPNDL812V, F815Y), VP2 (ERDQSTNG338F, DQ342H, VN344P, S368T, QNRSAP372G, L386F, RNSK438A, I465L), VP3 (A582S, PS597A, L601I, VL650I, LY672M),and VP4 (K273N), referring to SC1_LZ02.The remaining 13 mutations were located in the Leader (A26T, Q134R, ATV198E, DE199N, NKS202H, V204T), 2C (V1412I), 3A (TVI1464M, TVNS1577I), 3B (T1602A),and 3C (D1657E, T1658V, K1672R), referring to SC1_LZ02. Interestingly, SC1_LZ05 showed three aa deletions in structural proteins (431VKE, 496IH, and 595G) and SC1_LZ02 showed one aa deletion (1121E) in 2B. The MSA plots can be found in the Supplementary Materials.

By using RDP 4.101, a recombination event was detected in SC1_LZ02 with a single breakpoint, which was supported by seven methods including RDP, GENECONV, BootScan, MaxChi, Chimaera, SiScan, and 3Seq, and the recombinant score was 0.316. The breakpoint was located at nt 5173 of the whole genome in the putative major parental strain BRBV_6900 (GenBank Accession: MZ574106) and the minor parental strain BRBV_CHN1 (GenBank Accession: MT160419) (Figure 3).

## 4. Discussion

### 4.1. Prevalence of BRV

BRDC has long been a threat to the beef cattle breeding industry, and it has caused great economic losses all over the world [25]. There are multiple causative agents leading to BRDC, among which BRV is one of the most important pathogens. Based on the genomic characterization, BRV can be divided into BRAV and BRBV [26], and the two BRV genotypes have specific geographical distributions. Nevertheless, co-circulation of both genotypes can be observed. It is generally assumed that BRAV has a stronger replicative capability than BRBV, according to reports of the high viral load and frequent outbreaks [6,26], and it has caused significant losses in Japan and the United States [6,8]. Nowadays, all the existing BRV molecular assays are specifically targeting the 3D gene of the single BRV genotype [6,24,26,27], and the low sequence identity makes it difficult to detect simultaneous BRAV and BRBV using a single pair of primers. By sequence homology analysis, the average nt identities are 91.7% for BRAV 5′UTR and 95.67%for BRBV 3′UTR, showing a higher homology than that of 3D genes of both types of BRV (83.8% and 86.48%), suggesting that the UTRs are more suitable as detection targets. Thus, a duplex real-time RT-PCR assay targeting 5′UTR of BRAV and 3′UTR of BRBV was established, which enabled simultaneous detection of both types of BRV, and exhibited high specificity, sensitivity, and stability. In this study, the results of the prevalence investigation showed that 8/16 beef cattle farms in five provinces were positive for BRV, and the prevalence rates were 20.0~62.5% among these eight positive farms. Moreover, there were seven farms in four provinces that tested positive for BRAV, and three farms in three provinces tested positive for BRBV. It seemed that BRAV had a higher positive rate and wider geographical distribution than BRBV, and it has become the dominant circulating genotype of BRV in China. Interestingly, two cattle farms were observed with co-circulation of BRAV and BRBV, and five cattle were detected with co-infection of both genotypes. The co-infection of BRAV and BRBV has also been reported in the United States [6,26], and it might foster recombination and genetic evolution of the virus [28]. In conclusion, this study reported the presence and prevalence of BRAV in China, and it observed the co-circulation of BRAV and BRBV in cattle, which contributes to the further diagnosis and control of BRDC in China.

### 4.2. Genomic Characterization of BRAV

Until December 2022, there were 20 BRAV genomic sequences from six countries, including Germany, England, Japan, the United States, Mexico, and Canada, in the GenBank database. In this study, three near-full-length BRAV genomes were successfully obtained, and the phylogenetic analyses revealed that the three BRAVs consistently formed independent branches based on genomic sequences and each individual gene, suggesting that these three BRAVs were each undergoing an independent evolution. Notably, 11 unique aa mutations were identified in the three BRAVs compared to all known BRAV genomic sequences in GenBank, which localized in the Leader, VP1, VP3, 2A, 2B, 3A, 3C, and 3D proteins; however, the functional consequences of these unique aa mutations need further investigation.

As a major contributor to genetic diversity, homologous recombination becomes an important means for BRV evolution. In BRAV, recombination region shave been reported to locate in the Leader-VP3 and VP4-VP3 regions [22]. The recombination events were consistent in the three obtained BRAVs, and the recombinant regions were located in VP3 gene with breakpoints beginning at nt 2219 and ending at nt 2679, as was reported in the previous study [22], suggesting that recombination plays a role in the evolution of BRV. We further analyzed all 20 BRAV genomic sequences in the GenBank database and 9/20 of them were identified as recombinants (Supplementary Materials), suggesting that recombination is a common phenomenon in BRAV, although the biological significance of this needs further investigation.

### 4.3. Genomic Characterization of BRBV

In the present study, two near-full-length genomes of BRBV were successfully obtained and shared 79.2% nt and 85.3% aa identity with each other. The two strains were located on different phylogenetic branches and exhibited significant evolutionary divergence. Among them, SC1_LZ05 shared close genetic similarity with Chinese strain BRBV_CHN1 (97.1% nt identity and 99.3% aa identity) for which the genomic features had been well characterized [9], and no recombination event was observed in either of the two strains. Interestingly, SC1_LZ02 formed an independent clade and exhibited significant genetic distance from Chinese BRBV strains, exhibiting 33 unique aa mutations that localized in 9/12 proteins, including the Leader, VP1-4, 2C, 3A, 3B, and 3C proteins, compared to all available BRBV sequences in GenBank. Among these aa mutations, 19 mutations were located in the VP1, VP2, and VP3 proteins that make up the outer surface of the capsid [11,29] and contain major neutralizing sites [30,31]. Up to now, functions of VP1 and VP2 remain undetermined in BRV, while they have been well-studied in foot-and-mouth disease virus (FMDV), another well-known member of Aphthoviruses, and they are presumed to be related to antigenicity [32].The certain aa mutations in VP1 and VP2 are reported to cause immune escape of FMDV [32], and therefore it is reasonably speculated that the unique aa mutations in these two proteins of SC1_LZ02 might also cause immune escape, suggesting the importance of monitoring such BRV mutants. Interestingly, aa deletions were observed in VP2 (431VKE and 496IH) and VP3 (595G) of SC1_LZ05, which also presented in corresponding regions of FMDV type SAT2 (GenBank: MW715628.1), suggesting that aa deletion of structural proteins might play an important role in the evolution of Aphthoviruses. It was noteworthy that SC1_LZ02 showed an aa deletion in 2B protein (1121E), which seems not to occur in other Aphthoviruses, and the biological significance of these mutations needs further investigation.

The homologous recombination of BRBV was observed in a previous study, and the recombination regions had been reported to locate in the 2A-3C, Leader-3C, and VP1 regions [22], and the phenomenon was also found in this study. Notably, BRBV_CHN1 (GenBank Accession: MT160419) became the parental strain of SC1_LZ02, which confirmed that the BRBV strain previously identified on the southeastern coast subsequently was involved in the evolution of BRBV in China, and the recombination contributed to genetic diversification of the virus. Interestingly, the recombinant region of SC1_LZ02 expanded over the entire 3B-3D region, which was distinct from previous observations and might functionally affect RNA synthesis initiation [20,33], polyprotein cleavage [21], and genomic replication [22]. As the active center of 3D protein is not clear in BRV, the effects caused by aa mutations need further investigation. Remarkably, most of the aa mutations were located in non-recombinant regions of SC1_LZ02, illustrating that point mutations and homologous recombination collectively contributed to the evolution of BRV; however, the biological significance of these mutations and large segment displacement need further study.

## 5. Conclusions

In conclusion, this study reported the presence and prevalence of BRAV in China, which confirmed the co-circulation and co-infection of BRAV and BRBV. It was found that the recombination patterns were distinct from what was reported in a previous study, and it demonstrated the involvement of recombination in the evolution of BRBV. The present study provided genetic information for BRV, which serves the diagnosis and control of BRDC in China, and it contributes to a better understanding of the evolution of BRV.

## Figures and Tables

**Table 1:** Prevalence of BRV in nine provinces.

Province	Number of Samples	Number of Farms	Positive Rate of BRV	Positive Rate of BRAV	Positive Rate of BRBV	Positive Rate of Farms	95% CI of BRV Positive Rate
Shanxi	16	2	62.50% (10/16)	62.50% (10/16)	0.00% (0/16)	1/2	35.4–84.8%
Hebei	28	3	46.43% (13/28)	39.29% (11/28)	10.71% (3/28)	3/3	27.5–66.1%
Sichuan	44	3	38.64% (17/44)	27.27% (12/44)	20.45% (9/44)	2/3	24.4–54.5%
Jilin	9	1	33.33% (3/9)	0.00% (0/9)	33.33% (3/9)	1/1	7.5–70.1%
Jiangsu	15	1	20.00% (3/15)	20.00% (3/15)	0.00% (0/15)	1/1	4.3–48.1%
Shaanxi	15	2	0.00% (0/15)	0.00% (0/15)	0.00% (0/15)	0/2	0.0–21.8%
Qinghai	8	1	0.00% (0/8)	0.00% (0/8)	0.00% (0/8)	0/1	0.0–36.9%
Fujian	15	1	0.00% (0/15)	0.00% (0/15)	0.00% (0/15)	0/1	0.0–21.8%
Inner Mongolia	13	2	0.00% (0/13)	0.00% (0/13)	0.00% (0/13)	0/2	0.0–24.7%

**Table 2:** Amplification primers for BRAV and BRBV.

Primer Name	Forward Primer (5′ to 3′)	Reverse Primer (5′ to 3′)	Annealing Temperature (°C)	Amplified Fragment (nt)
BRAV-1	AAAGTGRCTAGTGGGCTG	CTGTCCCATAGGCTCCTC	50	889 (149–1038)
BRAV-2	TTTGCAACCCCTGATCC	TGTGTCCACCAGCAGTCAG	54	975 (973–1948)
BRAV-3	ATGTGGGCGCGTTCTC	AGCTCAAAATCTGGCCC	54	801 (1856–2657)
BRAV-4	GGATACAGATYTACCAGCTYG	TCAATTCAAGTATACCCTCAGTC	52	953 (2576–3529)
BRAV-5	GTTGAGACAAACCCYGG	CTGCCCRAGRTCGTCC	48	890 (3427–4317)
BRAV-6	CCCGSATGCTTACTGG	GCACCACTCTCAGTAGCCAC	55	957 (4208–5165)
BRAV-7	CAAAGCTGTGGRCAAYGC	GTAATGCTTGCTCTTCCG	55	874 (4976–5850)
BRAV-8	GGAAATGGRTACGCGTC	CCCTTAATGGTGTARCGC	53	961 (5704–6665)
BRAV-9	CCTGACGTGGACTGGC	GAGACACGGCCCGG	51	747 (6439–7186)
BRBV-1	CGGTTTTGCTGCTTTCAC	CARATGGTCTCRCGGTCC	54	1167 (28–1195)
BRBV-2	AGGCTGGWGTYTTCATGG	GTGATCRTCCATGTCDG	53	1006 (1042–2048)
BRBV-3	CTCTKTACCCMCACCA	GTCWGAAAARTAGTAGGTRCAWG	53	1144 (1957–3101)
BRBV-4	CACACTGAYGTTKCTTTYGC	RAAGAAACTCATAAGCCAYTG	52	1139 (2937–4076)
BRBV-5	GACACTAGARTYATGATGGAYA	CAAAGAGCCCRCACGG	52	960 (3843–4803)
BRBV-6	ATACCRCCRATGGCAGC	CCTATAACRAGGGTGTCAAAAC	54	1045 (4557–5602)
BRBV-7	GGSAACCCTCCAACTGAC	CRATAAGGTCATGTCTGCG	54	1014 (5433–6447)
BRBV-8	TACCGTCTYTGTGCTGCTG	ACGAAGGYACCTGTATGA	55	1157 (6270–7427)

The amplified fragments are referred to BRAV sd-1 (KP236128) and BRBV EC11 (EU236594).

## Data Availability

All data are available online. Sequences obtained in this study can be found in the NCBI GenBank database at https://www.ncbi.nlm.nih.gov/nucleotide/ assessed on 24 May 2022.

## Supplementary Materials

The following supporting information can be downloaded at: https://www.mdpi.com/article/10.3390/ani13020312/s1, SupplementaryMaterials.pdf: Phylogenetic trees for each gene of BRAV and BRBV; MSA plot of BRAV and BRBV; Recombination events of BRAV and BRBV.
